# Supplementary material for: Establishing pregnancy-specific haematological reference intervals in Ghana; a three-center cross-sectional study
Source: PLoS One. 2023 Feb 3;18(2):e0274422. doi: 10.1371/journal.pone.0274422 (PMC9897535; doi:10.1371/journal.pone.0274422)
Supplement: Attachment — Submitted filename: Comment to authors.docx [file pone.0274422.s003.docx]

In Table 4 (page 14), the 90% confidence interval for the upper limits of the following parameters do not seem technically or clinically possible: HCT (351), MCHC (418 and 223), RDW (447). I would suggest to remove these columns.
