# Supplementary material for: Establishing pregnancy-specific haematological reference intervals in Ghana; a three-center cross-sectional study
Source: PLoS One. 2023 Feb 3;18(2):e0274422. doi: 10.1371/journal.pone.0274422 (PMC9897535; doi:10.1371/journal.pone.0274422)
Supplement: Attachment — Submitted filename: Responses to reviwer comments.docx [file pone.0274422.s004.docx]

**RESPONSES TO REVIEWERS’ COMMENTS**

Establishing pregnancy-specific haematological reference intervals in Ghana; a three-center cross-sectional study (manuscript ID: PONE-D-22-04279)

| COMMENTS | RESPONSE | PAGE |
| --- | --- | --- |
| 1. In Table 4 (page 14), the 90% confidence interval for the upper limits of the following parameters do not seem technically or clinically possible: HCT (351), MCHC (418 and 223), RDW (447). I would suggest to remove these columns. | Table 4 has been revised to address this; a footnote “Note: the upper limit of the 90% CI of HCT, MCH, MCHC, and RDW for T2 are not indicated as they were very large to be considered technically feasible” has been added to highlight this change. | Page 14, line 281 – 282 |
| 2. A reference was added for the >13 cut-off, but there is insufficient rationale to justify its use. Please provide more in-depth rationale and discuss the impact of high HGB on pregnancy outcomes. | Manuscript has been revised to include additional information “Maternal haemoglobin and adverse birth outcomes have been shown to follow a U-shaped curve where both low and high gestational haemoglobin are associated with adverse pregnancy outcomes such as preterm, gestational diabetes, stillbirth, pre-eclampsia, and small-for-gestational age (1-3). Therefore, in accordance with previous meta-analyses report (4), intra-pregnancy Hb >13.0 g/dL was classified as high maternal haemoglobin to classify participants with high gestational haemoglobin.” Relevant references have also been cited. | Page 8, line 169 – 174 |
| 3. Both reviewers commented on the availability of quality control data for the hematology system in use. This needs to be included to ensure proper analytical performance was obtained throughout the study (i.e. was internal QC run?) | Per the prevailing protocols in each of the participating laboratory, control samples (low, normal and high) are run daily as quality control measure/check on analyser performance prior to assaying patient sample. Only when the respective haematology analyser passes the quality control tests is the analyser used to assay patient samples. Additionally, each of these daily quality control sample data (low, normal and high) are cumulatively evaluated monthly as a quality assurance measure to identify trends in analyser performance that may require immediate action. | Page 7, line 161 - 167 |
| 4. - In my opinion, graphs need to be provided by gestational age. Otherwise, it is very difficult to visualize the data and remark on observed trends. This would be very important to laboratories interpreting these findings. | This has been addressed in the revised manuscript; in figure 1A, [haemoglobin] is compared per trimester; figure 1C & 1F are specific trimester 2 [Hb] comparison per age category and participants’ place of residence; figure 1D & 1G are specific trimester 3 [Hb] comparison per age category and participants’ place of residence |  |

-

1.	Dewey KG, Oaks BM. U-shaped curve for risk associated with maternal hemoglobin, iron status, or iron supplementation. Am J Clin Nutr. 2017;106(Suppl 6):1694S-702S.

2.	Abeysena C, Jayawardana P, de ASR. Maternal haemoglobin level at booking visit and its effect on adverse pregnancy outcome. Aust N Z J Obstet Gynaecol. 2010;50(5):423-7.

3.	Díaz-López A, Ribot B, Basora J, Arija V. High and Low Haemoglobin Levels in Early Pregnancy Are Associated to a Higher Risk of Miscarriage: A Population-Based Cohort Study. Nutrients. 2021;13(5).

4.	Young MF, Oaks BM, Tandon S, Martorell R, Dewey KG, Wendt AS. Maternal hemoglobin concentrations across pregnancy and maternal and child health: a systematic review and meta-analysis. Ann N Y Acad Sci. 2019;1450(1):47-68.
