# Supplementary material for: Establishing pregnancy-specific haematological reference intervals in Ghana; a three-center cross-sectional study
Source: PLoS One. 2023 Feb 3;18(2):e0274422. doi: 10.1371/journal.pone.0274422 (PMC9897535; doi:10.1371/journal.pone.0274422)
Supplement: Attachment — Submitted filename: Responses to reviewer comments - PONE comments.docx [file pone.0274422.s002.docx]

**RESPONSES TO REVIEWERS’ COMMENTS**

Establishing pregnancy-specific haematological reference intervals in Ghana; a three-center cross-sectional study (manuscript ID: PONE-D-22-04279)

| Comment | Response | Page |
| --- | --- | --- |
| **EDITORIAL** |  |  |
| 1. Please, add line enumeration | Line numbers added |  |
| 2. As both reviewers point out there is an important amount of typos and orthographic issues to fix. | The manuscript has been revised to address these |  |
| 3. The authors declared that the dataset is fully available. I did not see such data. | The minimal data set underlying the results described in the manuscript has been uploaded as a Supporting Information file |  |
| 4. There are so many abbreviations. Please have a list of abbreviations at the end of the manuscript. | List of abbreviations included | Page 21;  Lines 147-155 |
| 5. The authors removed participants with malaria, and many other important conditions. However, nothing is mentioned about HIV (interestingly hepatitis B is). Why? | Hepatitis B infected participants’ data have been excluded; data has subsequently been re-analysed. All data from HIV positive pregnant women were not included in the initial data collection. | Page 6; Line 145 |
| 6. In the sample size subsection: - the last sentence starting at “The lower and upper reference…” should be removed. That is not part of sample size considerations. | “The lower and upper reference limits were determined non-parametrically using the 2.5^th^ and 97.5^th^ percentiles respectively” has been deleted from the “Sample size” section in the revised manuscript. | Page 6; Line 131-136 |
| 7. The “Reference interval estimation” subsection should be merged with the “Data management and data analyses” | The “reference interval estimation” section has been merged with the “Data management and data analyses section.” | Pages 8-9; Line 174 - 190 |
| 8. Data management and data analyses - Please cite the software used - For Excel, please add as well the version | The **Data management and data analyses** section of the manuscript has been revised to include “Data was double-entered and checked in a Microsoft Excel 2016 Spreadsheet. For statistical analysis, the Statistical Package for the Social Sciences (SPSS) version 22 (IBM Inc., USA), GraphPad Prism software version 8.0.2 (GraphPad Prism Inc., San Diego, USA) and Stata IC version 16.0 (Stata Corp, College Park, TX, USA) were utilized.” | Page 8;  Line 174 – 178. |
| 9. Table 1: - Please a row for overall total | Row totals have been calculated | Page 9; line 197-198 |
| 10. Table 2: - Please a row for overall total   - For hemoglobin classification, please add a footnote explaining the limits | Row totals have been calculated  Manuscript has been revised to include a footnote  “Anaemia was classified based on the trimester-specific cut-offs set by the World Health Organization; trimester 1 (Hb <11.0 g/dl), trimester 2 (Hb <10.5 g/dl), and trimester 3 (Hb <11.0 g/dl); in accordance with previous meta-analyses report (4), intra-pregnancy Hb >13.0 g/dL was classified as high maternal haemoglobin.” | Page 10; line 207-208  Line 209 - 211 |
| 11. Table 3: - Add a footnote explaining the abbreviations | Footnote defining abbreviations have been added. | Page 12; line 249 - 251 |
| 12. Figure 1: - It is dangerous the mixture boxplots and line plots with presumably errors whiskers (which are in fact percentiles). Please separate these and explain in the footnote how the errors in the line plots are computed | All graphs have been converted to Boxplots; a footnote “(All box plots were constructed with whiskers indicating minimum and maximum data points)” has been added. | Page 11; Line 228 – 231. |
| 13. **Figure 2:** - There are no dotted lines. There are dashed lines. | Figure 2 has been converted into a table (table 4) as suggested by both reviewer 1 & 2 | Page 13; line 254 - 268 |
|  |  |  |
| **REVIEWER 1** |  |  |
| Abstract |  |  |
| 1. CLSI should be written out in full since it is not a standard abbreviation | Done | Page 2; line 29 |
| 2. 2. Please clarify the version of the guidance document: is C28A2992 referring to C28-A3? | Yes. Please see reference | Page 2; line 38 |
| 3. 3. It is not clear why Hb >13 stated here and, in the discussion, should be considered as being high and linked to unfavorable pregnancy outcomes | This has been clarified under section “full blood count”; this is from a meta-analyses report by Young et al (2019) “Young MF, Oaks BM, Tandon S, Martorell R, Dewey KG, Wendt AS. Maternal hemoglobin concentrations across pregnancy and maternal and child health: a systematic review and meta-analysis. Ann N Y Acad Sci. 2019;1450(1):47-68.” | Page 7; Line 163-164 |
| 4. 4. It might be good to limit the number of decimal points for the various parameters as follows: Hb - 1, MCHC - 1, RBC - 2, MCH - 1, MCV - 1 MCH - 1, WBC - 1, and PLT - 0 (in the manuscript). | All decimals have been limited to 1 in all tables. |  |
| 5. Please indicate in the conclusion the comparison for which the determined reference intervals are lower | This is applicable to all the parameters analysed in the current study. |  |
| **Keywords** |  |  |
| 6. the keywords look a bit too long and repetitive, and could be revised to: Haematological; Reference intervals, Intra-pregnancy; Sub-Saharan Africa, pregnancy | Manuscript revised to reflect suggestions made by reviewer | Page 3; line 52-53 |
| **Introduction** |  |  |
| 7. CLSI is Clinical and Laboratory Standards Institute, and not Clinical Laboratory Standards Institute | Manuscript revised to read Clinical and Laboratory Standard Institute (CLSI). | Page 4; line 83 |
| 8. Please provide references for statements attributed to CLSI, and also references for previous research in Sub-Saharan Africa which authors indicate showed lower values | Done. |  |
| 9. Please note NCCLS is an older name of CLSI, kindly update to CLSI | Manuscript revised to uniformly read CLSI |  |
| 10. The last sentence of the introduction sounds like a conclusion and could be revised to explain the aim of the study. | The last section of the introduction has been revised to read “Since intra-pregnancy haematological reference intervals have not been explored within the sub-Saharan African region, we aimed to generate empirical baseline data to begin to address this knowledge gap, and stimulate discussion as well as further research that will ultimately lead to improved context-specific intra-pregnancy clinical decision-making process.” | Page 5; line 114 - 117 |
| **Methods** |  |  |
| 11. Please indicate the make and manufacturer of the haematology analyzers used for the analysis | The section has been revised accordingly to include “5-part Sysmex 20N haematology analyser (Sysmex Corp., Japan)” | Page 7; line 160 |
| 12. Please indicate Quality Assurance/Quality Control measures put in place to ensure reliable results | The “Data was double-entered and checked in a Microsoft Excel 2016 Spreadsheet” in the “**Data management and data analyses”** has been elaborated by adding “Data entry were at each study site was undertaken independently by two researchers after which they were validated by the research supervisor as a quality control process to ensure that data was accurately captured” to reflect QA procedures employed during data acquisition | Page 8; line175 – 178. |
| 13. Please give references for the sickling and G6PD tests | Reference provided in the revised manuscript “Barabara J. Bain IB, Michael A Laffan, S. Mitchell Lewis. Dacie and Lewis Practical Haematology: Elsevier Churchill Livingstone; 2011. 650 p.” |  |
| **Results** |  |  |
| 14. Reference values are supposed to be determined using apparently healthy individuals. Inclusion of the Hepatitis B positive individuals will affect the determined values. Please exclude these individuals from the calculations | Data from hepatitis B positive individuals have been excluded and data re-analysed. | Page 6; line 145 |
| 15. Presentation of the reference intervals in a tabular form will make it easier for the ready than the figures | All the reference intervals have been presented in tabular form **(see table 4)** | Page 14 |
| 16. The minimum of 120 individuals recommended by the CLSI for the determination of reference intervals is to make it possible to calculate 90% confidence intervals around the lower and upper values. The confidence intervals seem to be missing in the manuscript. | Manuscript has been revised to include the 90% confidence intervals for both the lower and upper limits; see table 4. | Page 14 |
| **Discussion, References** |  |  |
| 17. The discussion is well written but could be updated with more references comparing similar studies. | More references added |  |
| 18. the reference section looks too short. Kindly update. | References added |  |
| **REVIEWER 2** |  |  |
| **Abstract:** |  |  |
| 1. Suggest to remove "Deranged" and replace with "Abnormal"   - Suggest to include weeks of gestation for each trimester, space allowing | Done | Page 2; line 29  Page 2; line 35 – 36 |
| **Methods** |  |  |
| 1. Exclusion criteria: Please clarify whether individuals with acute illness at the time of collection and/or use of prescribed medication (e.g. thyroid supplementation) | Data from pregnant women with clinically diagnosed acute or chronic diseases were excluded from the study.  The only recorded medications the participants were on were the “routine pregnancy drugs” The inclusion criteria section has therefore been revised to include:  “All participants were on the following medications: folic acid (5 mg daily), oral iron tablets (200 mg three times daily) and oral multivitamin tables; these medications are generally called routine pregnancy drugs within the Ghana Health Services as they are part of the routine medical care for all pregnant women.” | Page 6; line 138 – 141. |
| Study participants: Please clarify whether iron supplementation was captured during assessment.  In addition, was any longitudinal assessment evaluated at the end of the pregnancy? | Iron supplementation is mandatory for all pregnant women in Ghana as part of routine care. This fact has been included in the  No | Page 6; line 138 – 141 |
| - Reference Interval Determination: Please add confidence intervals for each estimate. CLSI guidelines recommends reporting 90% CIs | Manuscript has been revised to include the 90% confidence intervals for both the lower and upper limits; see table 4. | Page 14 |
| **Experimental Protocols:** There is insufficient information in this section. For every assay, the manufacturer analyzers should be reported as well as their method (i.e. traceability) and performance throughout study duration (i.e. CV for QC levels).  In addition, multiple centers were included in this study - were any center-specific differences in methodology evaluated? | Each site uses 5-part Sysmex haematology analyser. Since this is a retrospective data that was retrieved from the hospital data management system, we were unable to collect the performance data of the analysers since the data was not prospectively collected.    We believe this would have been valid undertaking if the design for the study was a prospective one since this would have allowed us to interrogate the comparability of the laboratory results as well as inter- and intra-laboratory agreement. |  |
| - Data Analyses: CLSI guideline recommends using the Harris and Boyd method to test for partitions (i.e. gestational-age specific differences). Please clarify why MW and KW tests were selected. | The recommendation from Harris and Boyd was employed by comparing the standard deviations of parameters per trimester (table 3).  The MW and KW statistical procedures were only employed to test statistical differences between the haematological values. |  |
| In addition, please clarify if outliers were removed and whether normality of data were assessed prior to statistical testing. | The data was explored for outliers using the recommendation from the CLSI prior to analyses.  Data was assessed for normality before choosing non-parametric means was selected for the data selection. |  |
| **Results:** |  |  |
| Figure 1: Suggest to include these graphs for every parameter in supplemental methods. | Noted |  |
| **Table 1**: Please clarify if individuals were excluded on the basis of positive Hep B status. | Data from hepatitis B positive individuals have been excluded from the revised manuscript; see under the revised “Exclusion criteria section”. | Page 6; line 145. |
| **Table 3:** Suggest to include RIs in this table as well (or an additional table) rather than in the graphs. | The rational for including table 3 is to provide basis for why a separate or combined reference intervals should be used. The reference intervals are included in table 4. | Page 14 |
| In addition, was the differential evaluated? | No |  |
| **Discussion:** |  |  |
| The discussion mainly focuses on Hgb levels and their classification based on WHO standards. It should be highlighted that the WHO standards may not suit the ethnic population of Ghana and it is potentially not a public health problem. If no pregnant individuals or their neonates had serious complications, should this be considered abnormal? Suggest to include a comment on this. | We have acknowledged the cross-sectional nature of the study as a key limitation as we could not access pregnancy outcome data. | Page 19; line 374 – 386. |
|  |  |  |
|  |  |  |
