# Supplementary material for: Establishing pregnancy-specific haematological reference intervals in Ghana; a three-center cross-sectional study
Source: PLoS One. 2023 Feb 3;18(2):e0274422. doi: 10.1371/journal.pone.0274422 (PMC9897535; doi:10.1371/journal.pone.0274422)
Supplement: Attachment — Submitted filename: Point-by-point responses to reviewer comment 3.docx [file pone.0274422.s005.docx]

**Re**

| Comment | Responses | Page |
| --- | --- | --- |
| Editor comment |  |  |
| 1. Line 228 - I think the "semester" should be "trimester" | Correction effected | Page 11; line 230. |
| 2. For the prevalence of anaemia, please add 95% confidence interval (exact binomial confidence interval. In fact, this could be a separate figure or table. | Manuscript revised to include the 95% CI (exact binomial confidence interval) “Furthermore, whereas 46.5% of participants had low haemoglobin level and therefore anaemic, 9.3% had higher haemoglobin levels. When participants haemoglobin levels were considered per trimester, 36.4% (95% CI: 0.1719 – 0.5934), 31.6% (95% CI: 0.2487 – 0.3904) and 68.2% (95% CI: 0.5951 – 0.7601) respectively of participants in trimester 1, 2 and 3 were anaemic.”  However, since the main import of the manuscript is that using the Caucasian-centric reference intervals (which was the basis for the anaemia prevalence in table 2), may lead to some participants incorrectly classified as anaemic, we are of the opinion that constructing additional table/figure will not be necessary in this instance. | Page 10; line 216 - 220 |
| 3. The lines between 226 to 262 fail to describe the results properly. Rather than talking about the medians being compared per different groups, the authors focus only on p-values. | The description to the figure has been revised to include the respective median to enable the reader better appreciate the data. The revised manuscript thus read “Participants’ haemoglobin levels were compared with the demographic data (figure 1). Overall, the median haemoglobin significantly differed across the trimesters of pregnancy. The median haemoglobin was higher in trimester 1 (11.80 g/dl) but decreased progressively across trimesters 2 (11.00 g/dl) and 3 (10.50 g/dl) [fig 1A: p = 0.0232 (trimester 1 vs trimester 3); p = 0.0005 (trimester 2 vs trimester 3)]. There was a general trend toward higher median haemoglobin levels with advancing age of participants as the respective median haemoglobin levels for 14 – 19 years, 20 – 29 years, 30 – 39 years, and 40 – 49 years were 10.35 g/dl, 10.80 g/dl, 11.00 g/dl, and 11.10 g/dl [fig 1B; p = 0.049 (14 – 19 years vs 30 – 39 years group; p = 0.0278 (14 – 19 years vs 40 – 49 years group)]; a similar trend was also observed when data was explored for trimester 2 (figure 1C) and trimester 3 (figure 1D) although the differences did not reach statistical significance. Also, the median haemoglobin level was significantly higher in participants from Ashanti region (11.80 g/dl) compared to median haemoglobin level of participants from Eastern region (10.50 g/dL) or Upper Est region (10.30 g/dl) [figure 1E: p <0.0001 (Ashanti vs Eastern region); p <0.0001 (Ashanti vs Upper West region)]. Similarly, the median haemoglobin levels in trimester 2 was significantly higher in participants from Ashanti region (11.80 g/dl) compared to participants from Eastern (10.80 g/dl) or Upper West (10.30 g/dl) regions (figure 1F: p = 0.0031 vs Eastern region; p <0.0001 vs Upper West region). Moreover, the median haemoglobin in trimester 3 was higher in participants from Ashanti region (11.65 g/dl) compared to the Upper West (10.50 g/dl) region (figure 1G: p = 0.0240).” | Page 12; line 227 – 246. |
| Reviewer 1 |  |  |
| There are still very abnormal values indicated in Table 4 for the 90% CI for trimester 3 MCHC (223) and trimester 2 TWBC (87). As indicated in the earlier review comment, several of the upper values of the 90% CI are not clinically feasible and recommended the column could be removed. | Corrections effected | Page 15; line 284 - 289 |
|  |  |  |
